# Association of lactate to albumin ratio and bicarbonate with short-term mortality risk in patients with acute myocardial infarction

**DOI:** 10.1186/s12872-022-02902-4

**Published:** 2022-11-18

**Authors:** Jia-Liang Zhu, Hui Liu, Li-Li Wang, Xue-Hao Lu, Hai-Yan Yin, Jun Lyu, Jian-Rui Wei

**Affiliations:** 1 Department of Intensive Care Unit, The First Affiliated Hospital of Jinan University, Guangzhou, Guangdong 510630 China; 2 Guangzhou Women and Children’s Medical Center, Guangzhou Medical University, No. 9 Jinsui Road, Guangzhou, Guangdong 510220 China; 3 Department of Cardiology, Affiliated Hospital of Shandong University of Traditional Chinese Medicine, 16369 Jingshi Road, Jinan, Shandong China; 4 Department of Clinical Research, The First Affiliated Hospital of Jinan University, Guangzhou, Guangdong 510630 China

**Keywords:** Lactate/albumin ratio, Bicarbonate, Acute myocardial infarction, Risk of short-term mortality

## Abstract

**Background:**

Previous studies have indicated that the ratio of lactate/albumin (L/A) has predictive value for the prognosis of critically ill patients with heart failure. Some studies have also indicated that a low serum bicarbonate concentration is inversely related to the mortality risk of patients with cardiogenic shock. However, the value of bicarbonate and the L/A ratio for predicting the mortality risk of patients with acute myocardial infarction (AMI) is still unclear. We therefore conducted a retrospective study to research this problem.

**Methods:**

The subjects of this study were patients with AMI, and the data source was the Medical Information Mart for Intensive Care III database. The primary endpoint was 30-day all-cause mortality after admission. The Receiver operating characteristic (ROC) curve was used to compare the predictive value of L/A ratio, lactate and albumin for end-point events. The effects of different L/A ratio levels and different bicarbonate concentrations on 7-day and 30-day all-cause mortality were compared using Kaplan–Meier (K-M) curves. Hazard ratios for different L/A ratio and different bicarbonate concentrations were investigated using COX proportional hazards models.

**Results:**

The Area Under Curve (AUC) of L/A ratio, lactate, and albumin were 0.736, 0.718, and 0.620, respectively. (1) L/A ratio: The patients were divided into three groups according to their L/A ratio: tertile T1 (L/A ratio ≤ 0.47), tertile T2 (L/A ratio ≤ 0.97), and tertile T3 (L/A ratio > 0.97). The T2 and T3 groups had higher 30-day all-cause mortality risks than the T1 group. The restricted cubic spline (RCS) model indicated that there was a nonlinear relationship between L/A ratio and 30-day mortality (*P* < 0.05). (2) Bicarbonate concentration: The patients were also divided into three groups based on their bicarbonate concentration: G1 (22–27 mmol/L), G2 (< 22 mmol/L), and G3 (> 27 mmol/L). The G2 and G3 groups had higher 30-day all-cause mortality risks than the G1 group. The RCS model indicated that there was a nonlinear relationship between bicarbonate concentration and 30-day mortality (*P* < 0.05). The RCS model indicated that there was a nonlinear relationship between hemoglobin level and 30-day all-cause mortality (*P* < 0.05).

**Conclusion:**

L/A ratio and bicarbonate concentration and hemoglobin level have predictive value for predicting 30-day mortality in patients with acute myocardial infarction.

## Introduction

A previous study found that the hospital mortality rate of patients with acute myocardial infarction (AMI) is 5.1% [1]. Reportedly 5–15% of patients with AMI will develop cardiogenic shock (CS) [2]. Studies have shown that serum lactate is an independent predictor of in-hospital mortality in patients with ST-segment elevation myocardial infarction complicated with cardiogenic shock [3]. Tissue hypoperfusion is often present in patients with CS and is accompanied by increased lactic acid concentrations, and even metabolic acidosis. Acidosis generally manifests in two ways: increased lactate and decreased bicarbonate concentrations. Acidosis presents in the late stages of many diseases, and often indicates their poor prognosis. Early recognition of acidosis therefore has the potential to improve the prognosis of patients with acute myocardial infarction (AMI).

The increased risk of all-cause mortality was found to be related to low serum bicarbonate concentration in a cohort study of chronic kidney disease [4, 5]. Another study indicated that lower bicarbonate concentrations were associated with a higher mortality risk among patients with CS [6]. Previous research also indicated that low bicarbonate concentrations at ICU admission can accurately predict the mortality of patients with acute aortic dissection (AAD) [7].

Lactate is a product of anaerobic metabolism, which somewhat reflects tissue hypoperfusion. Lactate is a valuable parameter for circulatory failure diagnoses, treatment effect evaluations, and prognoses. However, increased lactate concentrations can be influenced by different factors, including seizures, cardiac arrest, diabetic ketoacidosis, burns, hepatic dysfunction, and metformin overdose [8]. The liver is also involved in the process of lactic acid metabolism. When the liver function is abnormal, it will affect the metabolism of lactic acid and further cause the accumulation of lactic acid. Albumin is produced by the liver and can reflect the state of liver function. Higher lactate levels or lower albumin levels can directly and indirectly indicate lactate metabolic dysfunction, respectively. Therefore, the L/A ratio can better reflect the situation of lactate metabolism. Additionally, albumin concentrations are affected by many factors such as nutritional status and nephrotic syndrome. Using only albumin concentrations for predictions may therefore have limitations.

Studies have shown that the L/A ratio has a predictive value for the mortality of patients with heart failure [9]. In predicting the outcome of severe sepsis, the predictive effect of L/A ratio is better than that of lactic acid alone [10]. Gharipour et al. also found that the L/A ratio can be used as an effective predictor for the risk stratification of critically ill patients [8]. In addition, studies have shown that in invasively treated patients with acute coronary syndromes, a decrease in hemoglobin level of more than 3 g/dl is associated with an increased risk of one-year all-cause mortality [11].

The value of the L/A ratio and the bicarbonate concentration and hemoglobin level in predicting the prognosis of patients with AMI is still unclear. We therefore retrospective explored the predictive value of the L/A ratio and the bicarbonate concentration and hemoglobin level for 30-day mortality in patients with AMI.

## Methods

### Data source

The source of the data for this retrospective study was the Medical Information Mart for Intensive Care III (MIMIC-III) database. The MIMIC-III is an intensive care database created by the Massachusetts Institute of Technology. It includes more than 40,000 patients admitted to clinical care units from 2001 to 2012. It also contains comprehensive patient information, including laboratory examinations, patient characteristics at admission, medication status, and all medical records [12]. After completing the relevant NIH course and examinations, and we successfully obtained permission to access the data (no. 45848365). This study also passed a review by the ethics committee of our hospital.

### Participant selection

Patients with AMI were excluded if they had the following characteristics: (1) < 18 years old, (2) > 90 years old, (3) missing lactic and albumin data, or (4) missing bicarbonate data.

### Data extraction

Structured Query Language was used to extract indicators within 24 h of admission from the MIMIC-III database. These indicators included basic information, comorbidities, vital signs, and other variables. Comorbidities included congestive heart failure, cardiac arrhythmias, valvular disease, peripheral vascular disease, hypertension, chronic pulmonary disease, depression, simple diabetes, complicated diabetes, renal failure, and liver disease. We also extracted laboratory data including hemoglobin, potassium, bicarbonate, ALT, AST, CK, CK-MB, sodium, hematocrit, lymphocyte, PLT, PT, WBC, SBP, DBP, MBP, heart rate, respiration rate, body temperature, SpO_2_, and glucose [13].

### Primary endpoints

The endpoint of this study was 30-day all-cause mortality.

### Statistical analysis

Normally distributed continuous variables are represented by mean and standard deviation values, and Analysis of Variance (ANOVA) were used to compare measured data between groups. Categorical variables are expressed as frequency and percentage values, The χ2 or Fisher exact test was used to compare enumeration data between groups. We included the ratio of lactate to albumin, lactate, and albumin into multivariate model, respectively, and compared the predictive value of end-point events by the Receiver operating characteristic (ROC) curve analysis. Area Under Curve (AUC) ≥ 0.5 and < 0.7: low predictive value; AUC ≥ 0.7 and less than 0.9: moderate predictive value; AUC ≥ 0.9: high predictive value). In addition, we further compared the AUC value of lactate and L/A ratio. A two-sided *P* value of < 0.05 was considered statistically significant in all analyses.

#### (1) Lactate/albumin ratio

All baseline variables were divided into three groups based on their L/A ratio tertiles. Kaplan–Meier (K-M) curves were used to calculate survival probabilities and to compare the differences between the three groups. Cox proportional-hazards models were used to test the associations between different groups and results (with the first group as the reference), and the results were expressed as hazard ratios (HRs) and 95% confidence intervals (CIs). The COX hazard proportional regression model that only included the L/A ratio was model I. To reduce errors, all continuous variables, sex, race and comorbidities were included in the multivariate Cox proportional-hazards models (model II). Restricted cubic splines (RCS) were used to obtain the relationship between the L/A ratio and 30-day all-cause mortality risk.

#### (2) Bicarbonate

The normal range of the bicarbonate concentration is 22–27 mmol/L, and patients within this range were defined as group 1 (G1, *n* = 385). Those with concentrations < 22 mmol/L were defined as group 2 (G2, *n* = 365), and those with concentrations > 27 mmol/L were defined as group 3 (G3, *n* = 92). The K-M curve was used to calculate the survival probabilities of G1, G2, and G3. We used Cox proportional-hazards models to determine the relationship between bicarbonate concentrations and the results (with G1 as the reference group), and outcomes were presented as HRs and 95% CIs. The COX hazard proportional regression model that only included the bicarbonate was model 1. All continuous variables, sex, race and comorbidities were included in the multivariate Cox proportional-hazards models (model 2). RCSs were used to obtain the relationship between bicarbonate concentration and 30-day all-cause mortality risk.

#### (3) Hemoglobin

RCSs were used to obtain the relationship between hemoglobin levels and 30-day all-cause mortality risk.

## Results

**Fig. 1:**
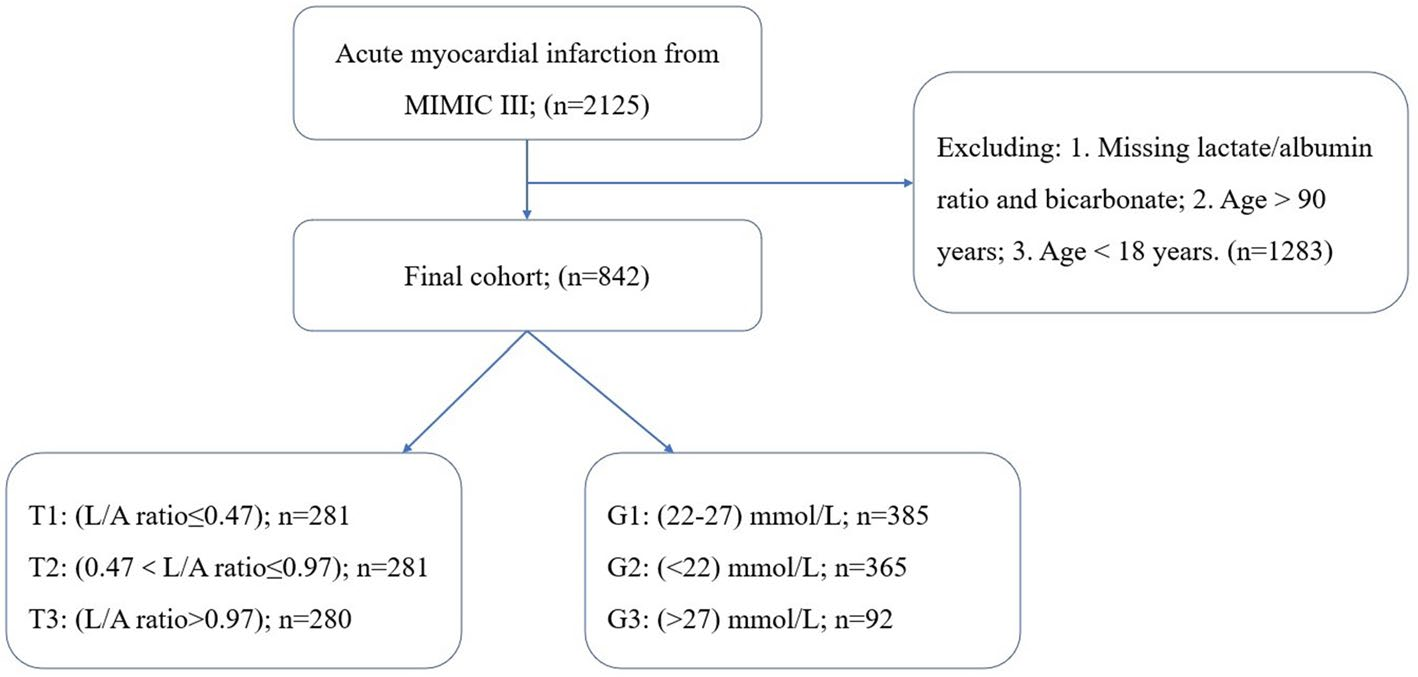
Study flow diagram depicting exclusion criteria and outcomes

Overall, 2,126 patients with AMI were extracted from the MIMIC-III database, of which 1,283 patients lacked lactate, albumin and bicarbonate. Therefore, 843 patients were finally included in this study, of which 205 (24.3%) died within 30-day of admission (Fig. 1).

Patients were divided into three groups based on their L/A ratio: tertile 1 (T1), tertile 2 (T2), and tertile 3 (T3). Table 1 compared the baseline characteristics in the three groups. As the L/A ratio increased, so did age, breathing rate, and heart rate. SBP, DBP, MBP, body temperature, and SpO_2_ decreased as the L/A ratio increased. Potassium, ALT, AST, CK, PT, and WBC increased, while bicarbonate, lymphocyte, hematocrit, hemoglobin, and PLT decreased as the L/A ratio increased. The incidence rates of congestive heart failure, arrhythmia, simple diabetes, and liver disease increased with the L/A ratio. The incidence rates of valvular disease, peripheral blood vessel disease, hypertension, chronic lung disease, diabetic comorbidities, renal failure, and depression decreased as the L/A ratio increased.

**Table 1:** The baseline characteristic of the population

Variables	Lactate/albumin ratio tertiles			*P*-value
	T1 ( L/A ratio ≤ 0.47)	T2 (0.47 < L/A ratio ≤ 0.97)	T3 (L/A ratio > 0.97)	
Number of patients	(*N* = 281)	(*N* = 281)	(*N* = 280)	
Gender (%)				0.063
Male	192 (68.3)	189 (67.3)	167 (59.6)	
Female	71 (30.1)	81 (34.3)	97 (40.9)	
Age (years)	67.9 (12.9)	66.7 (12.8)	68.6 (12.6)	0.203
Race (%)				0.285
White	180 (64.1)	191 (68.0)	168 (60.0)	
Black	10 (3.6)	11 (3.9)	22 (7.9)	
Other	91 (32.4)	79 (28.1)	90 (32.1)	
Hematocrit (%), mean (SD)	35.7 (6.09)	35.9 (6.57)	35.2 (6.61)	0.378
Lymphocyte (%), mean (SD)	13.3 (7.93)	12.8 (8.51)	13.4 (9.88)	0.713
Hemoglobin (g/dL), mean (SD)	11.5 (1.90)	11.9 (2.11)	11.4 (2.33)	0.006
Potassium (mmol/L), mean (SD)	4.18 (0.720)	4.18 (0.813)	4.28 (0.954)	0.259
ALT (U/L), mean (SD)	51.4 (66.8)	127 (566)	137 (327)	0.015
AST (U/L), mean (SD)	107 (138)	231 (864)	240 (618)	0.018
CK (IU/L), mean (SD)	827 (1650)	887 (1810)	1450 (6140)	0.101
CKMB (IU/L), mean (SD)	56.1 (118)	71.1 (243)	60.2 (107)	0.550
Sodium (mmol/L), mean (SD)	138 (4.59)	138 (4.59)	138 (5.04)	0.318
Bicarbonate (mmol/L), mean (SD)	23.1 (4.47)	22.8 (4.07)	19.9 (5.17)	< 0.001
PLT (K/uL), mean (SD)	249 (98.0)	254 (115)	236 (108)	0.135
PT (s), mean (SD)	14.9 (5.10)	15.4 (7.39)	15.7 (4.16)	0.277
WBC (K/μL), mean (SD)	11.6 (5.63)	13.4 (6.55)	14.6 (7.54)	< 0.001
Heart Rate(beats/min) mean (SD)	82.2 (13.3)	89.2 (15.8)	88.9 (16.4)	< 0.001
SBP (mmHg), mean (SD)	114 (14.3)	111 (15.2)	106 (13.8)	< 0.001
DBP (mmHg), mean (SD)	59.0 (10.2)	60.0 (10.3)	57.8 (9.74)	0.029
MBP(mmHg), mean (SD)	77.1 (9.81)	77.5 (9.99)	75.1 (10.6)	0.011
Respiration rate (beats/min), mean (SD)	18.2 (3.51)	19.5 (4.33)	19.4 (4.25)	< 0.001
Body temperature(℃), mean (SD)	36.9 (0.647)	37.0 (0.742)	36.7 (0.985)	< 0.001
SpO2 (%), mean (SD)	97.5 (1.94)	97.3 (2.35)	96.9 (4.62)	0.080
Glucose (mg/dL), mean (SD)	145 (39.9)	161 (61.1)	175 (58.2)	< 0.001
Congestive heart failure, n (%)	133 (47.3)	151 (53.7)	135 (48.2)	0.259
Cardiac arrhythmias, n (%)	138 (49.1)	156 (55.5)	162 (57.9)	0.099
Valvular disease, n (%)	56 (19.9)	44 (15.7)	41 (14.6)	0.206
Peripheral vascular, n (%)	28 (10.0)	35 (12.5)	24 (8.6)	0.310
Hypertension, n (%)	175 (62.3)	138 (49.1)	149 (53.2)	0.006
Chronic pulmonary, n (%)	58 (20.6)	57 (20.3)	48 (17.1)	0.515
Diabetes uncomplicated, n (%)	66 (23.5)	67 (23.8)	74 (26.4)	0.678
Diabetes complicated, n (%)	31 (11.0)	23 (8.2)	13 (4.6)	0.002
Renal failure, n (%)	62 (22.1)	50 (17.8)	47 (16.8)	0.238
Liver disease, n (%)	16 (5.7)	23 (8.2)	34 (12.1)	0.024
Depression, n (%)	14 (5.0)	16 (5.7)	12 (4.3)	0.746

*Abbreviations*: *ALT* Alanine aminotransferase, *AST* Aspartate aminotransferase, *CK* Creatine kinase, *CKMB* Creatine Kinase Myocardial Band, *PLT* Platelets, *PT* Prothrombin time, *WBC* White blood cell, *SBP* Systolic blood pressure, *DBP* Diastolic blood pressure, *MBP* Mean blood pressure, *Spo2* Blood oxygen saturation

### ROC curve analysis

**Fig. 2:**
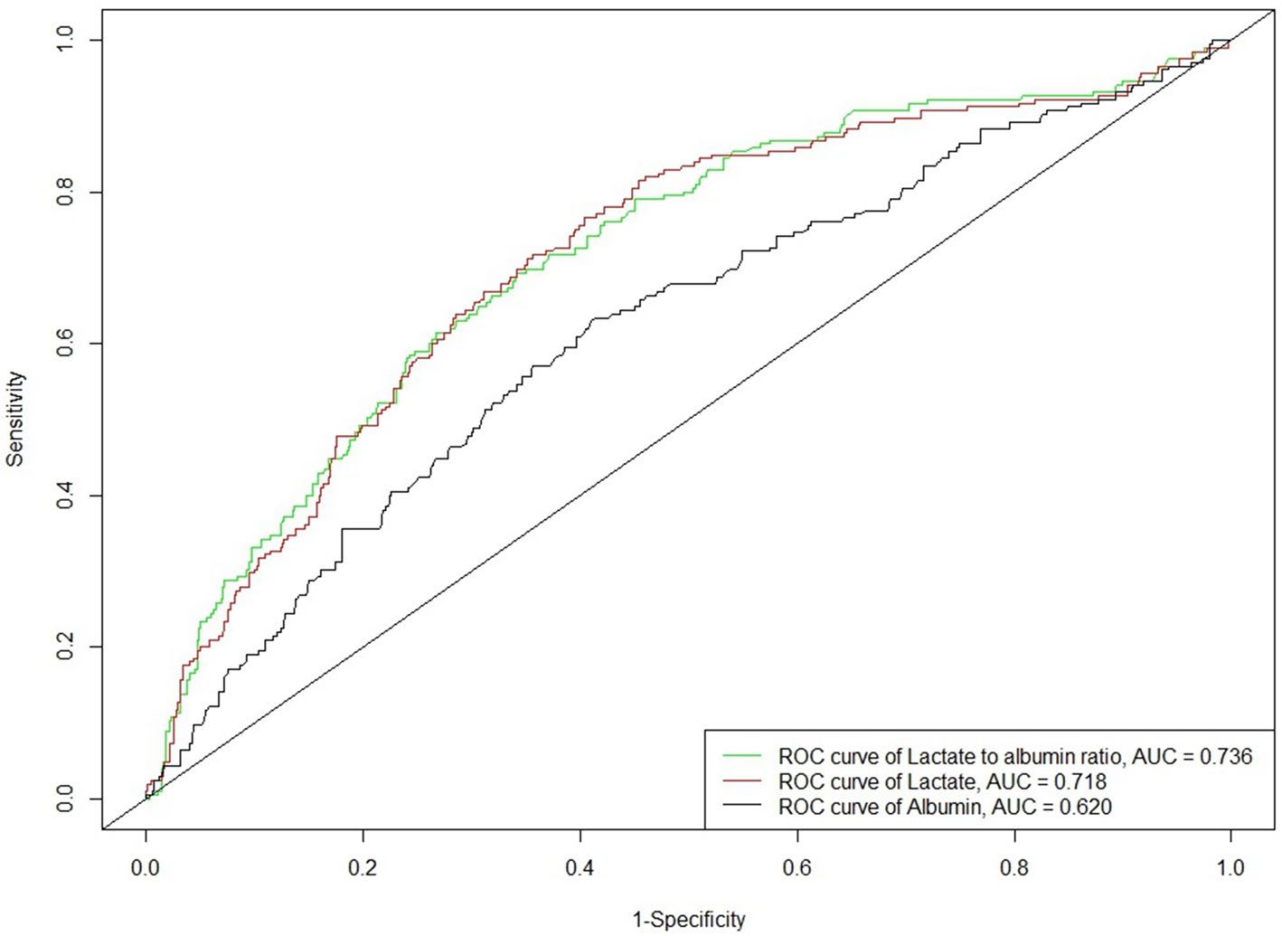
Receiver operating characteristic (ROC) curve results of Lactate to albumin, Lactate and Albumin

The AUCs for L/A ratio, lactate, and albumin were 0.736, 0.718, and 0.620, respectively (Fig. 2). The AUC of L/A ratio and lactate both exceeded 0.7, indicating that both variables have good predictive value. The comparison of AUC of L/A ratio and lactate showed that the *P*-value < 0.05. The AUC of L/A ratio was the highest, indicating that the predictive value of L/A ratio was better than that of lactate and albumin.

### Lactate/albumin (L/A) ratio

**Fig. 3:**
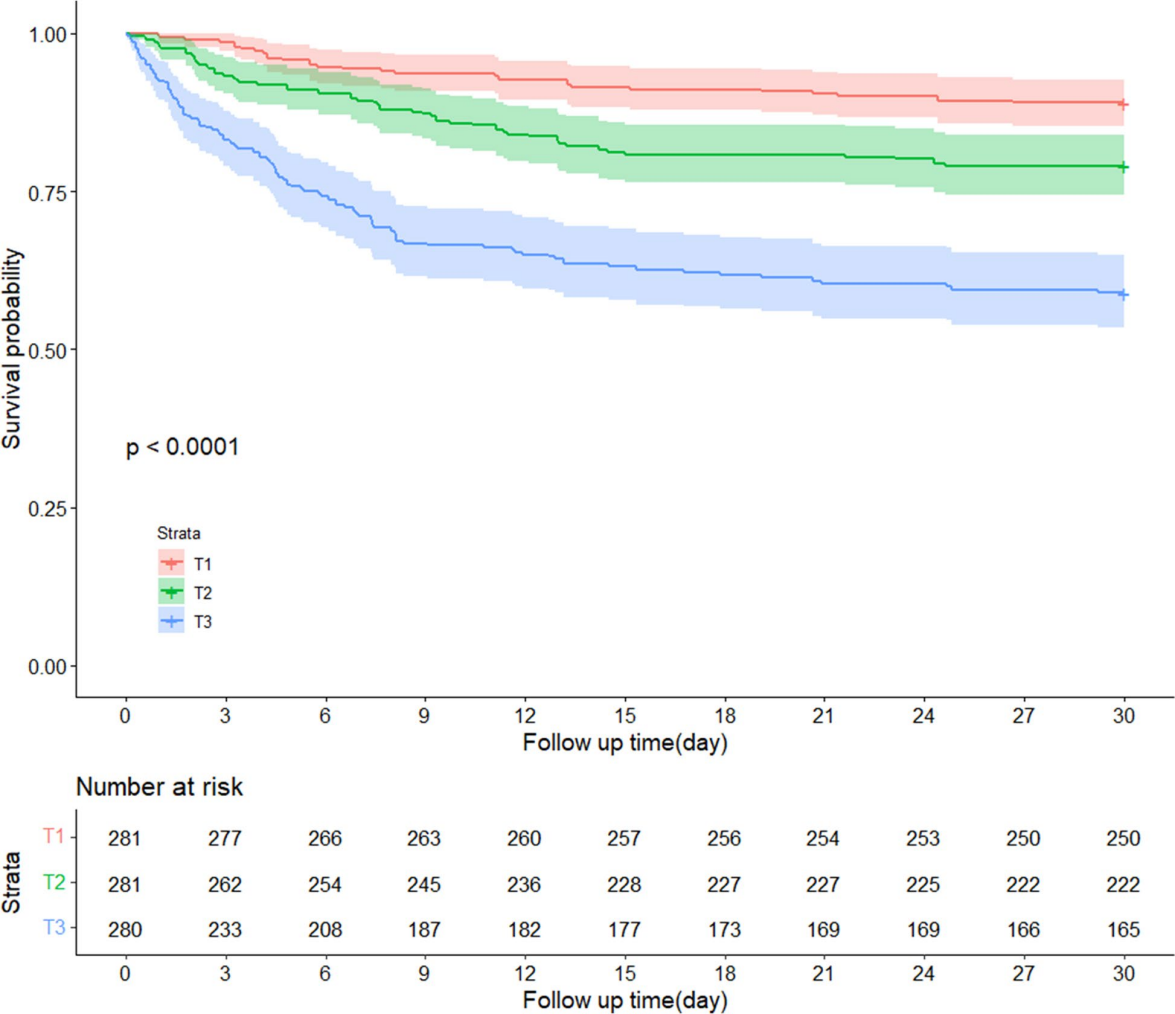
Kaplan–Meier (K-M) curve showing the survival probability of 30-day by lactate/albumin (L/A) ratio tertile among patients with AMI

**Fig. 4:**
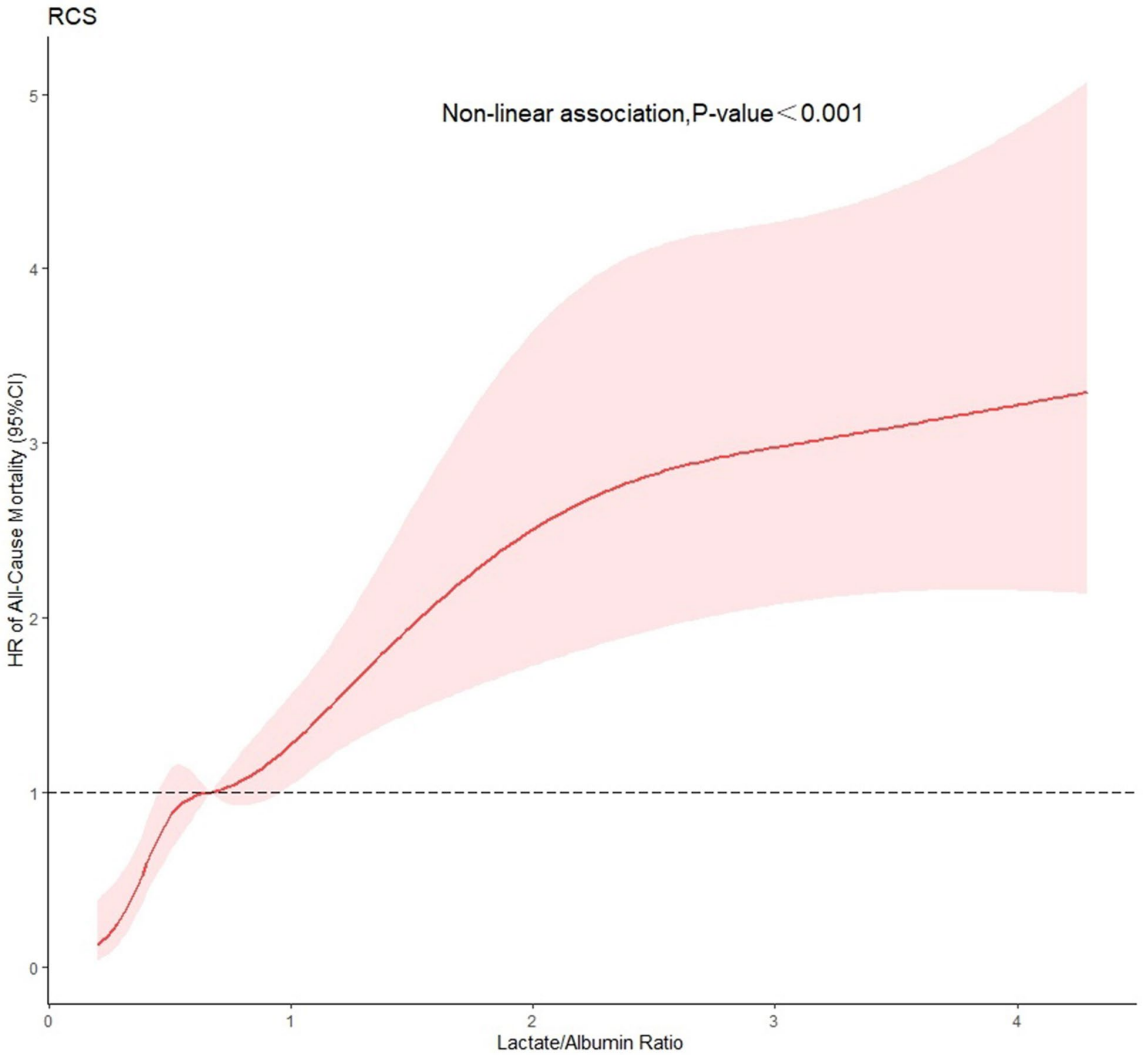
RCS curves showing association between lactate/Albumin (L/A) ratio and 30-day all-cause mortality

The K-M curve indicated a statistically significant difference between the survival probabilities of the three groups, and the log-rank test indicated *P* < 0.001 (Fig. 3). In model I, the mortality risks for patients in T2 and T3 were higher than in T1 (HR = 2.178, 95% CI = 1.336–3.549; and HR = 5.078, 95% CI = 3.247–7.941; respectively). In model II, the mortality risks for patients in T2 and T3 were higher than in T1 (HR = 1.73, 95% CI = 1.057–2.841; and HR = 3.251, 95% CI = 2.056–5.143; respectively) (Table 2). The differences between T1 and T2 (*P* < 0.05) and T1 and T3 (*P* < 0.05) were statistically significant (*P* < 0.05). RCS indicated that there was a nonlinear relationship between L/A ratio and 30-day mortality (*P* < 0.05) (Fig. 4).

**Table 2:** Hazard ratio (95% confidence interval) of 30-day all-cause mortality according to tertiles of L/A ratio

Variable	L/A ratio tertiles			
	Model I		Model II	
	HR (95% CI)	*P*-value	HR (95% CI)	*P*-value
T1	1.0 (Reference)		1.0 (Reference)	
T2	2.178 (1.336–3.549)	0.002	1.733 (1.057–2.841)	0.029
T3	5.078 (3.247–7.941)	< 0.001	3.251 (2.056–5.143)	< 0.001

Model I: univariable analysis, Model II: multivariable analysis

### Bicarbonate concentration

**Fig. 5:**
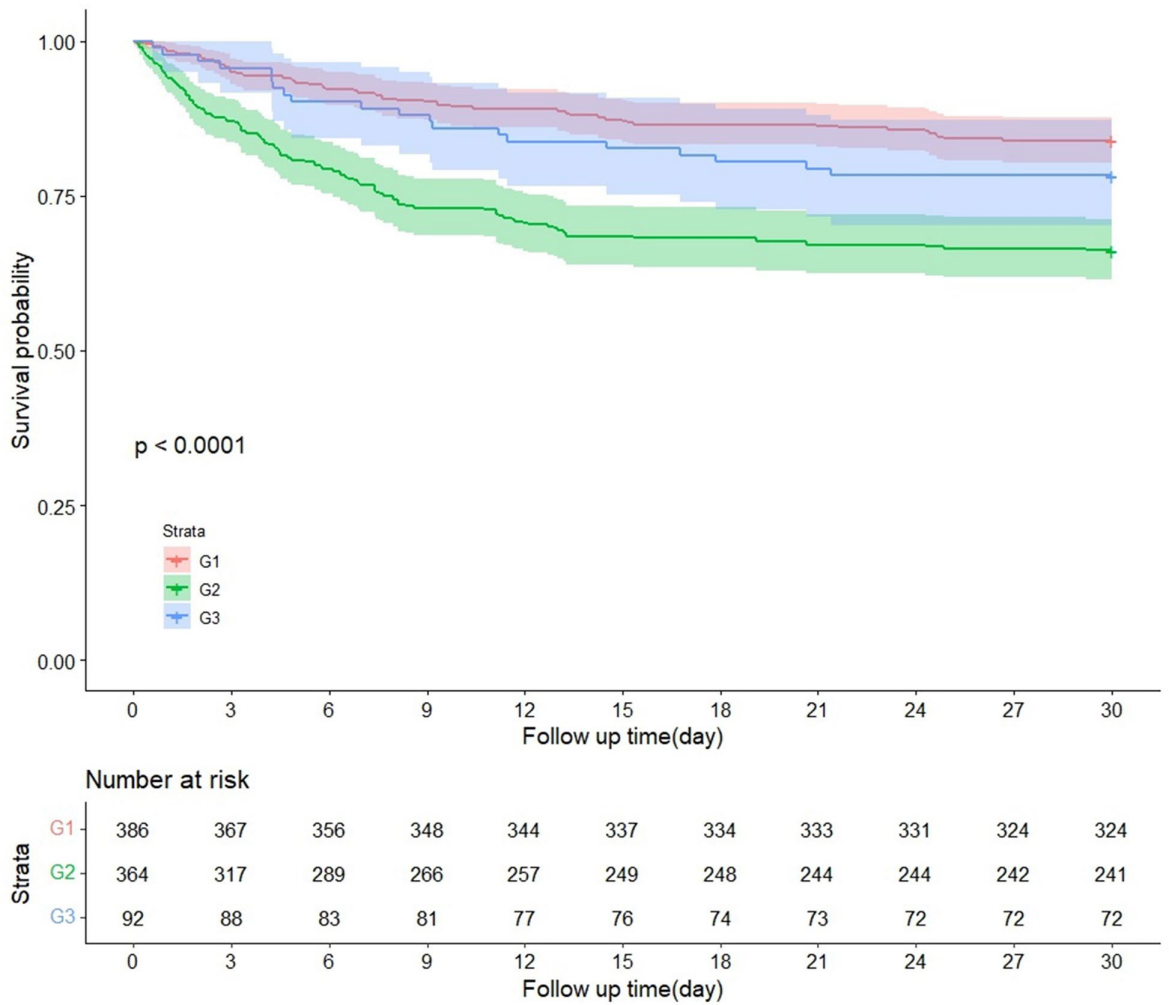
Kaplan–Meier (K-M) curve showing the survival probability of 30-day by G1 and G2 and G3 among patients with AMI

**Fig. 6:**
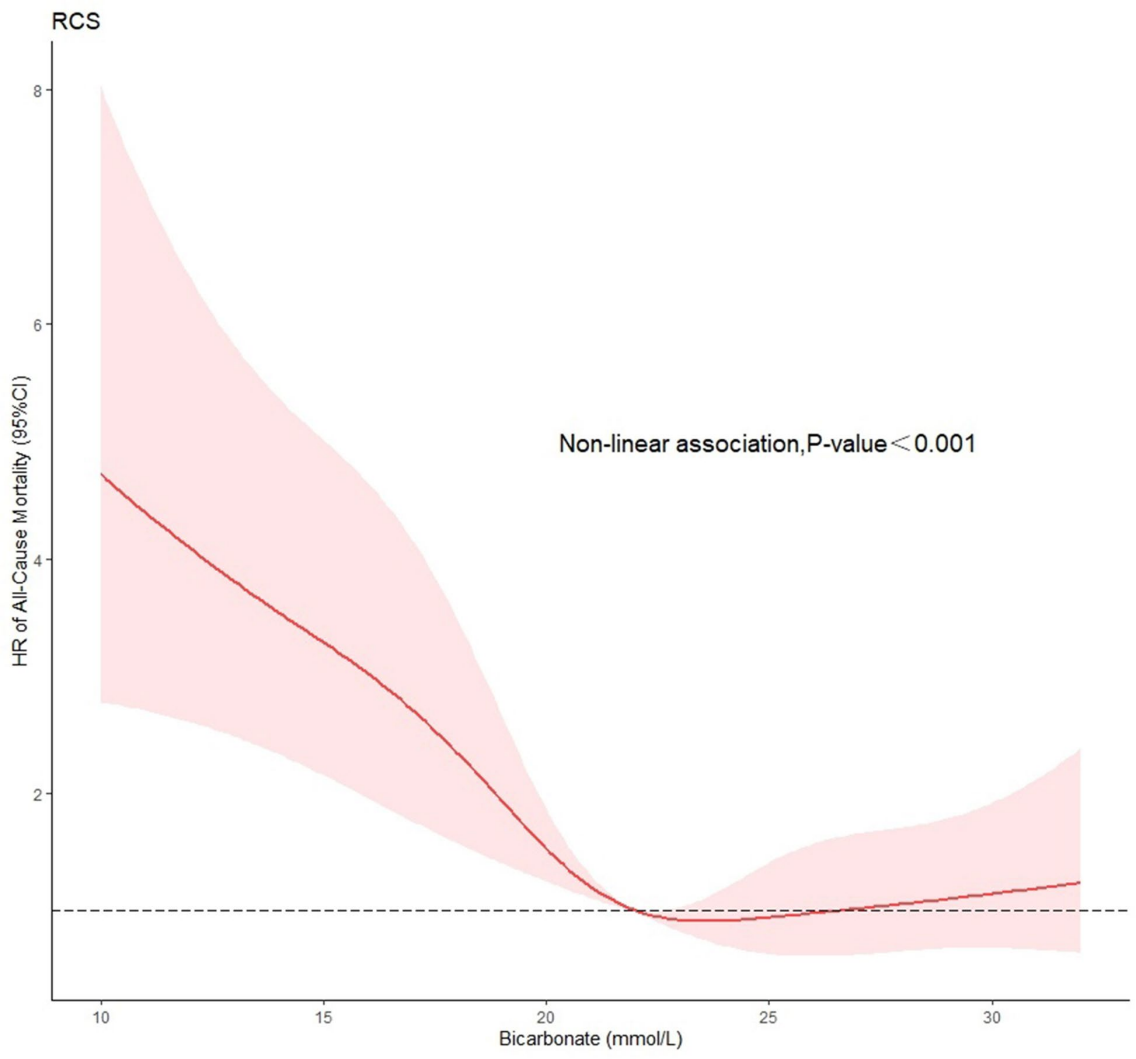
RCS curves showing association between bicarbonate concentration and 30-day all-cause mortality

The K-M curve indicated a statistically significant difference between the survival probabilities of the three groups, and the log-rank test indicated *P* < 0.001 (Fig. 5). In model 1, the 30-day mortality risks for patients in G2 and G3 were higher than in G1 (HR = 2.624, 95% CI = 1.959–3.514; and HR = 1.549, 95% CI = 0.971–2.471; respectively). In model 2, the mortality risks for patients in G2 and G3 were higher than in G1 (HR = 1.934, 95% CI = 1.425–2.624; and HR = 1.694, 95% CI = 0.996–2.551; respectively) (Table 3). The difference between G1 and G2 was statistically significant (*P* < 0.05), but that between G1 and G3 was not statistically significant (*P* > 0.05). The RCS model indicated that there was a nonlinear relationship between bicarbonate concentration and 30-day all-cause mortality (*P* < 0.05) (Fig. 6).

**Table 3:** Hazard ratio (95% confidence interval) of 30-day all-cause mortality according to G1 and G2 and G3

Variable	Bicarbonate concentration			
	Model 1		Model 2	
	HR (95% CIs)	*P*-value	HR (95% CIs)	*P*-value
G1	1.0(Reference)		1.0(Reference)	
G2	2.624(1.959—3.514)	< 0.001	1.934(1.425–2.624)	< 0.001
G3	1.549(0.971—2.471)	0.066	1.694(0.996–2.551)	0.052

Model 1: univariable analysis, Model 2: multivariable analysis. G1: 22–27 mmol/L, G2: < 22 mmol/L, G3: > 27 mmol/L

### Hemoglobin level

**Fig. 7:**
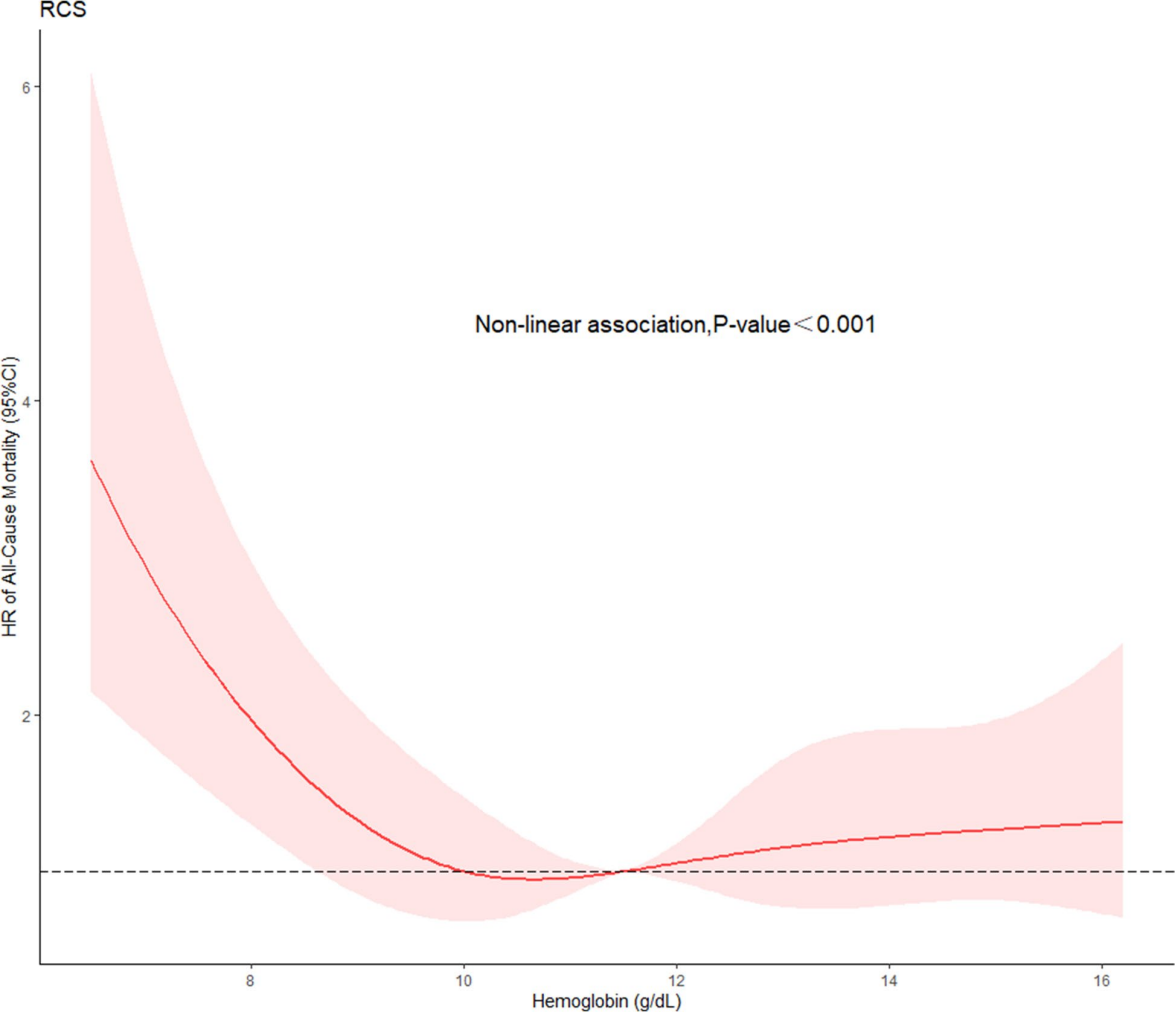
RCS curves showing association between hemoglobin level and 30-day all-cause mortality

The RCS model indicated that there was a nonlinear relationship between hemoglobin level and 30-day all-cause mortality (*P* < 0.05) (Fig. 7).

## Discussion

The K-M curve indicates that the mortality risk increased with the L/A ratio, and also that a lower bicarbonate concentration was indicative of a lower survival rate of patients with AMI. These findings suggest that excessive acid production in the body or decreased ability to remove acid will reduce the survival rate of patients with AMI. Our results indicate that the L/A ratio and bicarbonate concentrations measured on admission are meaningful in independently predicting the mortality of patients with AMI. The results from the RCS model indicated a nonlinear relationship between the L/A ratio and 30-day all-cause mortality risk. It also suggests that bicarbonate concentration has a nonlinear relationship with 30-day all-cause mortality risk. Notably, 30-day mortality risk increased with the L/A ratio. In addition, when the bicarbonate concentration was < 22 mmol/L, 30-day mortality risk increased as the bicarbonate concentration decreased.

Both increased lactate and decreased bicarbonate concentrations can promote acidosis. Previous studies have indicated that metabolic acidosis can reduce myocardial contractility, change systemic vascular resistance, and reduce adrenoreceptor response, leading to circulatory shock aggravation, promoted cell hypoxia, and poor tissue perfusion [6]. Additionally, regarding acidosis, potassium can be transferred from cells to the extracellular fluid and cause hyperkalemia, which increases the likelihood of various types of arrhythmia. Increased L/A ratio or decreased bicarbonate concentration should therefore be taken seriously, and the acidic environment of the extracellular fluid should be corrected as soon as possible in order to improve the prognosis.

While lactate has a good predictive value, explaining this relationship is complicated because other diseases may also cause elevation of serum lactate [14–19]. The L/A ratio can not only reflect the lactate level, but also reflect the body's ability to degrade lactate to a certain extent. The lactate concentrations of some high-risk patients are normal, which may cause underestimations of their mortality risks. Additionally, albumin has predictive value for the mortality of critically ill patients in APACHE II scores. However, plasma albumin concentrations may be affected by liver function, malnutrition, and nephrotic syndrome [10]. A previous study showed that an elevated C-reactive protein/albumin ratio was an independent predictor of risk of all-cause mortality in patients with ST-segment elevation myocardial infarction (STEMI) undergoing primary percutaneous coronary intervention (pPCI), suggesting that albumin alone is a predictor of poor performance [20]. A study exploring the relationship between serum uric acid/albumin ratio (UAR) and no-reflow in STEMI patients showed that UAR had better predictive power than albumin [21]. Using albumin as part of the L/A ratio to reflect liver function status increased its effectiveness in the prognosis of patients with AMI [10]. Changes in lactate and albumin are two mechanisms that work in opposite directions. When either lactate or albumin is changed, the L/A ratio can be changed. Therefore, the L/A ratio can accurately identify the patient's condition changes [10]. Regarding hepatic dysfunction, the ability to eliminate lactate will be reduced, and failure to timely eliminate lactate usually indicates a poor prognosis [22]. The L/A ratio is a combination of lactate and albumin concentrations, and its use as a prognostic factor in patients with AMI is a good choice.

The acid–base balance in the human body can be reflected by serum bicarbonate concentrations. Wigger et al. found that the baseline serum bicarbonate concentration was negatively correlated with the mortality risk among patients with CS [6], which was a similar result to ours. Our study indicates that low bicarbonate concentrations (< 22 mmol/L) are negatively correlated with 30-day mortality: as bicarbonate concentrations decreased, the mortality risk increased. Mortality risk was the lowest at bicarbonate concentrations in the range of 22–27 mmol/L. Early mortality in patients with AMI is high, and timely monitoring of baseline serum bicarbonate concentrations and implementation of interventions may help improve patient outcomes.

Our study showed that when the hemoglobin level was below 10 g/dL, the risk of mortality in patients with AMI increased as the hemoglobin level decreased. Decreased hemoglobin levels can reduce the oxygen content of arterial blood. There is an imbalance between myocardial oxygen demand and oxygen consumption, which may lead to adverse effects in patients with AMI.

Studies have shown that in STEMI patients, the Intermountain Risk Score was not inferior to Thrombolysis in Myocardial Infarction and Global Registry of Acute Coronary Events scores for predicting total mortality [23]. We hope that in future studies, combining L/A ratio, bicarbonate and hemoglobin, we will further explore a new scoring system for patients with AMI to assess prognosis.

## Conclusion

The L/A ratio was positively correlated with the 30-day all-cause mortality risk of patients with AMI, and the mortality rate increased with the L/A ratio. For bicarbonate concentrations < 22 mmol/L, it was inversely associated with 30-day mortality risk in AMI patients. Lower bicarbonate concentrations associated with higher 30-day mortality risk. When the hemoglobin level was less than 10 g/dl, the hemoglobin level was inversely associated with the 30-day risk of all-cause mortality in patients with AMI. Therefore, we believe that the use of L/A ratio and bicarbonate concentration and hemoglobin level as predictors of 30-day mortality in AMI patients is a good choice.

## Study limitations

Due to database limitations, this study has some limitations. First, variables with large missing proportions, such as troponin, killip class status and left ventricular ejection fraction, were deleted in this study. Second, only the primary values of L/A ratio and bicarbonate were extracted for analysis. In the future, we hope to extract data from multiple measurements and perform trajectory analysis to study the relationship between variable dynamics and prognosis.

## Data Availability

The data that support the findings of this study are available from https://mimic.mit.edu/, but restrictions apply to the availability of these data, which were used under license for the current study, and so are not publicly available. Data are however available from the authors upon reasonable request and with permission of National Institutes of Health.

## Abbreviations

MIMIC III: Medical Information Mart for Intensive Care III
ALT: Alanine aminotransferase
AST: Aspartate aminotransferase
CK: Creatine kinase
CKMB: Creatine Kinase Myocardial Band
PLT: Platelets
PT: Prothrombin time
WBC: White blood cell
SBP: Systolic blood pressure
DBP: Diastolic blood pressure
MBP: Mean blood pressure
Spo2: Blood oxygen saturation
